# Anti-*Toxoplasma gondii* effects of XYP1-derived peptides and regulatory mechanisms of XYP1

**DOI:** 10.1186/s13071-024-06455-7

**Published:** 2024-09-04

**Authors:** Jing Li, Kaijuan Wu, Xiaohua Liu, Dongqian Yang, Jing Xie, Yixiao Wang, Kang Liu, Zheng Wang, Wei Liu, Liping Jiang

**Affiliations:** 1 Department of Parasitology, School of Basic Medical Sciences, Central South University, Changsha, 410013 Hunan China; 2 China-Africa Research Center of Infectious Diseases, Central South University, Changsha, 410013 Hunan China; 3 Department of Vascular Surgery, The Third Xiangya Hospital, Central South University, Changsha, 410013 Hunan China; 4 Hunan Key Laboratory of Traditional Chinese Veterinary Medicine, Hunan Agricultural University, Changsha, 410128 China

**Keywords:** XYP1-derived peptides, Toxoplasmosis, Anti-*Toxoplasma gondii* activity, Transcriptome analysis

## Abstract

**Background:**

Toxoplasmosis, caused by *Toxoplasma gondii* , poses serious health issues for humans and animals. Individuals with impaired immune systems are more susceptible to severe toxoplasmosis. Pregnant women infected by *T. gondii* can face the possibility of birth defects and miscarriages. While pyrimethamine and sulfadiazine are commonly used drugs in clinical practice, concerns over their side effects and resistance are on the rise. A spider peptide XYP1 isolated from *Lycosa coelestis* had potent anti-*T. gondii* effects, but it had a high synthesis cost and strong cytotoxicity.

**Methods:**

This study intended to modify XYP1 for producing derived peptides via amino acid truncation and substitution. The anti-*T. gondii* effect was evaluated by trypan blue staining assay and killing experiment of RH strain tachyzoites. The CCK8 and hemolysis assays were used to compare their safeties. The morphological changes of *T. gondii* were observed by scanning electron microscope and transmission electron microscope. In addition, the mechanism of XYP1 against *T. gondii* through RNA-sequencing was further explored.

**Results:**

In vivo and in vitro experiments revealed that XYP1-18 and XYP1-18-1 had excellent anti-*T. gondii* activity with lower cytotoxicity and hemolysis activity than XYP1. XYP1, XYP1-18, and XYP1-18-1 were able to disrupt the surface membrane integrity of *T. gondii* tachyzoites, forming pores and causing the disruption of organelles. Furthermore, RNA-sequencing analysis indicated that XYP1 could stimulate the host immune response to effectively eliminate *T. gondii* and lessen the host’s inflammatory reaction.

**Conclusions:**

XYP1-18 had lower cytotoxicity and hemolysis activity than XYP1, as well as significantly extending the survival time of the mice. XYP1 played a role in host inflammation and immune responses, revealing its potential mechanism. Our research provided valuable insights into the development and application of peptide-based drugs, offering novel strategies and directions for treating toxoplasmosis.

**Graphical Abstract:**

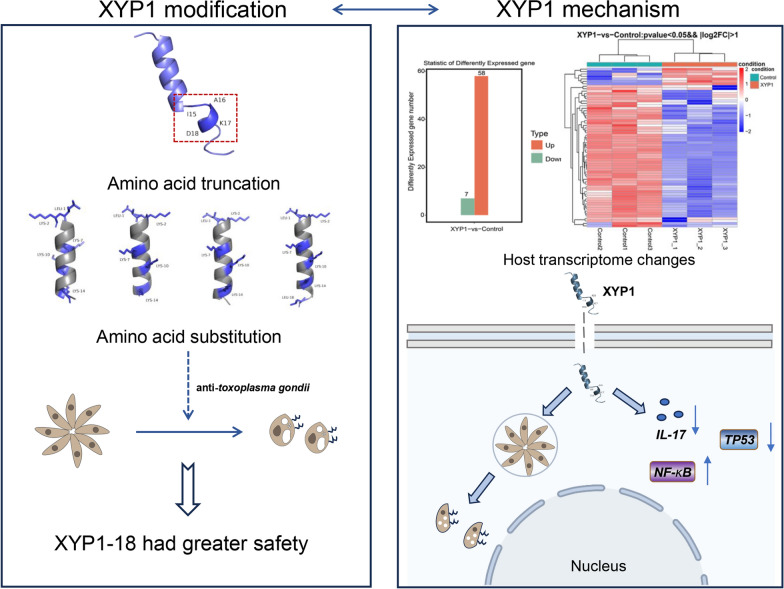

**Supplementary Information:**

The online version contains supplementary material available at 10.1186/s13071-024-06455-7.

## Background

*Toxoplasma gondii* (*T. gondii*) is an important opportunistic pathogen belonging to the phylum Apicomplexa. *T. gondii* can cause toxoplasmosis, which is a zoonotic parasitic disease affecting both humans and animals [1]. Globally, approximately one-third of the population is estimated to be infected with *T. gondii* [1, 2]. In immunocompromised patients, *T. gondii* infection can lead to significant morbidity and mortality. Currently, drugs such as pyrimethamine combined with sulfadiazine, trimethoprim combined with sulfamethoxazole, and spiramycin are mainly used in clinical treatment of toxoplasmosis [3, 4]. While these treatments can alleviate symptoms and control the disease, the limitations and side effects of drug treatment need to be taken into account [5]. *T. gondii* also showed increasing resistance to sulfadiazine and pyrimethamine [6]. Therefore, it is crucial to explore new treatment methods and drugs for the effective management of toxoplasmosis.

In recent years, peptide drugs have emerged as a novel therapeutic approach with various applications such as antiinflammatory, antitumor, and antimicrobial properties, making them valuable in treating a wide range of diseases [7–9]. Peptides offer the advantage of specific targeting toward diseases while reducing the complexity of toxic side effects and drug metabolism, thus becoming a new focus in drug research for treating multiple diseases. Studies have identified peptides with anti-*T. gondii* activity. For instance, cal14.1a was a peptide extracted from *Conus californicus*, which can reduce the invasion and proliferation of *T. gondii* in host cells [10]. Additionally, longicin P4, an alkaline peptide extracted from *Haemaphysalis longicornis*, had shown efficacy in limiting the growth of *T. gondii* [11]. HPRP-A1 and HPRP-A2, α-helical cationic peptides extracted from *Helicobacter pylori*, can effectively reduce the survival rate of *T. gondii* tachyzoites and inhibit their adhesion and invasion of macrophages [12]. These indicated that peptides still hold great potential and application space in the fight against *T. gondii* infection.

Our earlier studies have indicated that a novel spider polypeptide XYP1 derived from the venom of *Lycosa coelestis* suppressed the invasion and proliferation of *T. gondii* and extended the survival time of mice infected with *T. gondii* [13]. Compared with the conventional drug sulfadiazine, the peptide XYP1 showed superior therapeutic efficacy and fewer side effects, thus holding significant promise for the treatment of *T. gondii* infection. However, limitations such as strong cytotoxicity and high synthesis cost restricted its clinical application.

Peptide truncation can not only save the synthesis cost, reduce reaction time, and enhance efficiency, but also can alter the bioactivity, stability, hydrophilicity, hydrophobicity, and toxicity of peptides. The beneficial effects of peptide truncation have been reflected in arasin 1 and linear chicken β-defensin-4 [14–16]. The distribution of hydrophobic and hydrophilic residues is a crucial factor influencing the activity of antimicrobial peptides (AMPs), with hydrophilic residues typically concentrated near the membrane surface of AMPs, while hydrophobic residues are distributed internally. This distribution pattern facilitates the interaction of AMPs with the cell membrane, so as to enhance their bactericidal activity [17, 18].

In this study, XYP1 was truncated and replaced with amino acids to produce eight derived peptides. Two peptides, XYP1-18 and XYP1-18-1, with high anti-*T. gondii* activity were selected. XYP1-18 and XYP1-18-1 exhibited lower cytotoxicity and hemolysis compared with XYP1. Further in vivo and in vitro experiments indicated that XYP1-18 had similar activity as XYP1. The three peptides can damage the surface membrane and internal structure of *T. gondii* tachyzoites to different degrees. Additionally, we discovered that XYP1 can protect the host by reducing the inflammatory response and inducing immune response during *T. gondii* infection. In conclusion, our strategy may provide more insights and possibilities for peptide drug design and development, offering a novel approach for utilizing peptides to combat *T. gondii* infection in the future.

## Methods

### Parasites and cell culture

Tachyzoites of the RH-GFP-TgAtg8 and RH wild-type *T. gondii* strains were transmitted in human foreskin fibroblasts (HFFs). HFFs were growth in Dulbecco’s modified Eagle’s medium (DMEM, Gibco, USA) attached with 1% antibiotics (10 mg/mL streptomycin solution, 25 μg/mL amphotericin B, and 10,000 U/mL penicillin) (Sangon Biotech, China) and 10% fetal bovine serum (FBS, Invitrogen, USA) at 37 ℃ with the concentration of 5% CO_2._ The different *T. gondii* strains were maintained in HFFs with DMEM, 2% FBS, and 1% antibiotics in the same environment [19].

### Animals

The 6–8-week-old female BALB/c and Kunming mice used in this study were purchased from the Department of Laboratory Animals, Central South University in China.

### Sequence and structure analysis of polypeptides

The various characteristics of polypeptides including isoelectric point, net charge, and average hydrophilicity were analyzed on the website (https://www.expasy.org/) [20]. The tertiary structure of polypeptides was predicted by I-TASSER's online tool (http://zhanglab.ccmb.med.umich.edu/I-TASSER/) [21]. The spiral round figure was constructed by Heliquest online website (http://heliquest.ipmc.cnrs.fr/) [22].

### Chemical synthesis and identification of XYP1-derived peptides

The derived peptides were synthesized by Fmoc solid phase polypeptide synthesis method [23]. It mainly included the following steps: deprotection of Fmoc group, coupling reaction, washing, detection using the Kaiser method, and cleavage. High performance liquid chromatography (HPLC) technique (Shimadzu, Japan) was used to purify peptides, and electrospray ionization mass spectrometry (ESI–MS) (Shimadzu, Japan) was used to identification of derived peptides.

### Evaluation of anti*-toxoplasma gondii* effect

When *T. gondii* died, trypan blue was able to cross its cell membrane and enter the interior, making it blue. Trypan blue exclusion test was undertaken as mentioned before [24]. Firstly, 10 μM different XYP1-derived peptides were prepared, incubated with *T. gondii* for 2 h, and then the trypan blue dye was added. After 3–5 min, the survival rate of *T. gondii* was observed by optical microscope and the mortality rate was calculated.

The tachyzoites of RH-GFP-TgAtg8 strain did not fluorescein after death, which can be used to determine the difference in the anti-*T. gondii* activity of XYP1 and its derived peptides; 10 μM different derived peptides were incubated with tachyzoites of RH-GFP-TgAtg8 strain, respectively, and the fluorescence intensity and area were observed under fluorescence microscope (Leica DM IL LED, Germany).

### Hemolysis and cytotoxicity assays

The hemolytic activity of peptides was determined by the amount of hemoglobin released from lysed human red blood cells [25]. Normal human blood was mixed 1:1 with Aldrin’s fluid, followed by centrifuging, and phosphate buffered saline (PBS) was added to make 1% red blood cell resuspension. The 50 μL resuspension was mixed with 50 μL PBS, 1% Triton X-100, 70% DMSO (Sigma, USA), and different concentrations of polypeptides, then incubated at 37 ℃ for 30 min. After centrifugation, the OD_540_ value of the supernatant was measured by Microplate reader (Agilent, USA) and the hemolysis rate was calculated.

The CCK-8 method [26] was employed to assess the effect of different peptides on cytotoxicity, and 100 μL of cell culture solution containing 10 μM different peptides were added to each of the 96-well plates. After 24 h, 10 μL CCK-8 solution (APExBIO, USA) was added and incubated for another 2 h. The OD_540_ value was then measured and the cell mortality was calculated.

### Survival assay

The anti-*T. gondii* effect of peptides in vivo was investigated through a mouse survival experiment [27]. The experiment was divided into a PBS control group and different polypeptide groups. Each group of mice was intraperitoneally injected with 1000 RH strain tachyzoites. After 4 h, the pre-prepared 4 mg/mL peptides were injected into the mice. The survival time of mice in each group was recorded and analyzed statistically.

### Electron microscopy technology

The scanning electron microscope (SEM) was used to observe the surface structure of *T. gondii*, while the transmission electron microscope (TEM) can reflect the morphological changes of *T. gondii* inside the cell [28]. The sample preparation process for SEM included: the preparation process of SEM consists of fixing the *T. gondii* sample with 4% glutaraldehyde solution and staying overnight at 4 ℃. A series of gradient dehydration treatments are performed, followed by dehydration in anhydrous ethanol. After dehydration, the tachyzoites were dried and coated, followed by SEM observation (Hitachi S-3400N, Japan).

TEM steps mainly include fixing, rinsing, dehydration, infiltration, embedding, etc. After the *T. gondii* samples were fixed in 4% glutaraldehyde, they were washed with a cacodylate buffer, and 1% osmium tetroxide was used to fix the sample at 4 ℃ for 2 h. The fixed samples were then washed and subjected to a series of gradient dewatering treatments before being embedded in epoxy resin and polymerized at 60 ℃. After polymerization, the sample was sliced and double stained. Finally, the internal structure of *T. gondii* tachyzoites treated with different polypeptides was observed by TEM (Tecnai G2 Spirit TWIN, USA).

### RNA-seq analysis

The control group consisted of HFF cells infected with *T. gondii* without XYP1, and the experimental group was treated with XYP1 for 8 h. Total RNAs were extracted from the control and experimental groups and reverse to cDNA, with three replicates per group. After the steps of end repair, A-tailing, and adapter ligation, the samples were proceeded with polymerase chain reaction (PCR) amplification and Illumina transcriptome sequencing in the OE biotech Co., Ltd. (Shanghai, China). Trimmomatic software [29] was used to filter the raw data to obtain high-quality data information. Subsequently, the filtered data was aligned and assembled using HISAT2 [30] and StringTie [31] tool. The number of reads mapped to each gene in each sample was calculated.

DESeq software [31] was used to screen differentially expressed genes, and the screening conditions for differential genes were fold change > 2 and *P* value < 0.05. To further understand the function of differentially expressed genes, cluster analysis, gene ontology (GO) functional enrichment analysis [32] and Kyoto Encyclopedia of Genes and Genomes (KEGG) pathway enrichment analysis [33] were conducted on the selected genes. Additionally, the PPI network of differentially expressed proteins was constructed by the STRING website, and hub genes were identified by Cytoscape software.

### Quantitative real-time PCR (qRT-PCR)

The total RNAs from different samples were extracted using the classical Trizol method. The PerfectStart Uni RT&qPCR Kit (TransGen Biotech, China) was used to efficiently reverse transcribe RNA into cDNA and synthesize the first strand. The qRT-PCR reaction system contains cDNA temple, forward and reverse primers, double stranded DNA fluorescent dye (SYBR Green I), DNA polymerase, dNTP and buffer. The PCR reaction was performed using a two-step amplification procedure: 94 ℃ for 30 s (pre-denaturation) followed by 40 cycles of 94 ℃ for 5 s and 60 ℃ for 30 s. Finally, the relative expression of each gene was calculated by 2^−∆∆Cq^ method.

### Statistical analysis

The quantitative data was analyzed with the method of two-tailed Student’s *t*-test or two-way analysis of variance (ANOVA) with Tukey’s multiple comparisons in GraphPad 8.4 software. A *P*-value < 0.05 was considered to have a significant difference.

## Results

### Sequence analysis and structural modification of XYP1

**Fig. 1:**
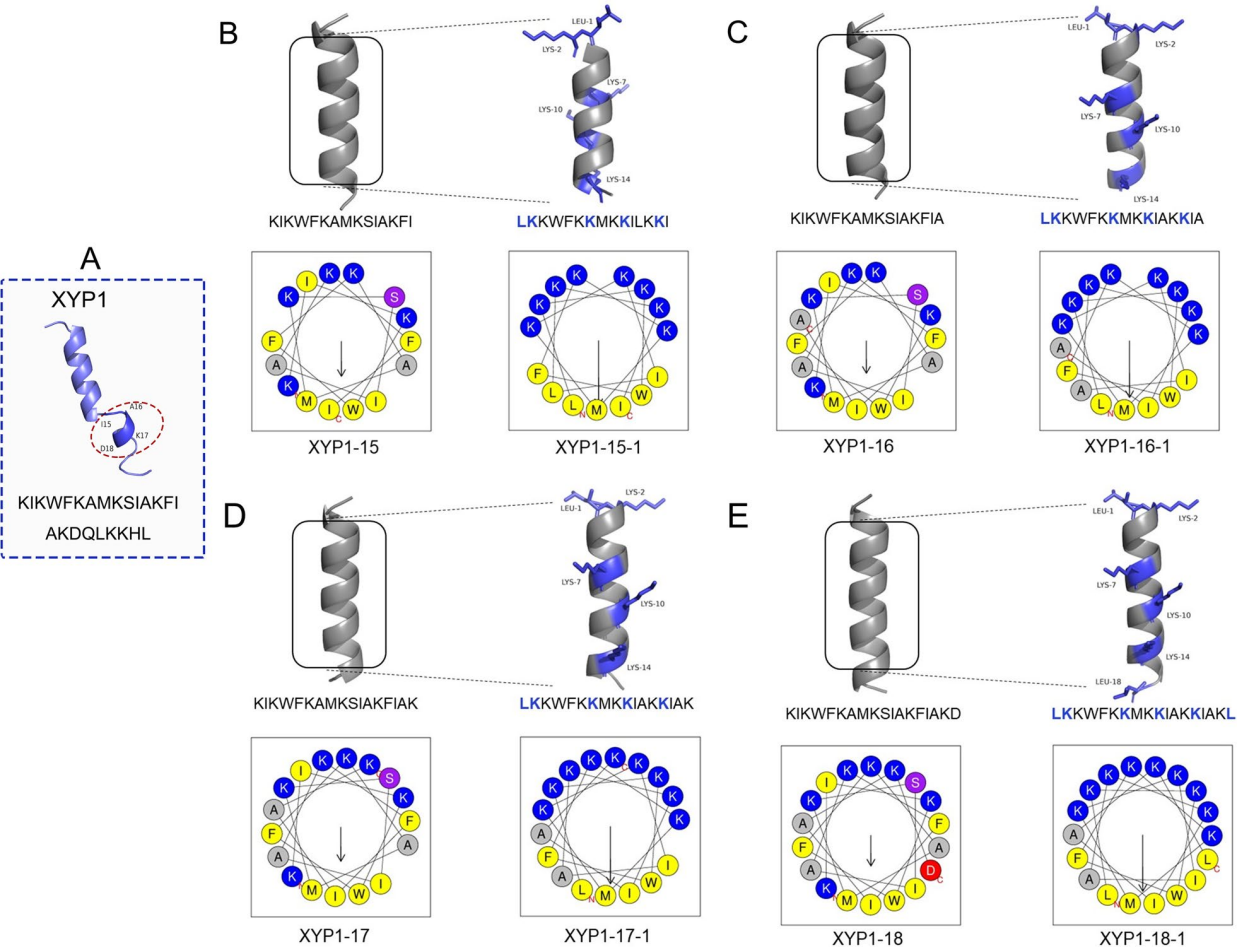
Structure diagram of XYP1 and its derived peptides. **A** The predicted tertiary structure diagram of XYP1, the dotted red boxes are the amino acids intended to be truncated. **B** The whole diagram is divided into four small pictures, with the left being the structure diagram and spiral wheel diagram of XYP1-15, and the right being the schematic diagram of XYP1-15-1; the residues after amino acid replacement of XYP1-15 are marked in blue. **C** The left diagram is the structure diagram and spiral wheel diagram of XYP1-16, and the right diagram exhibits XYP1-16-1; the residues after amino acid replacement of XYP1-16 are marked in blue. **D** The diagram on the left describes the structure and properties of XYP1-17, and the right diagram represents XYP1-17-1; blue marks the amino acids that have been replaced. **E** The figures on the left and right show the structure and properties of the derived peptides XYP1-18 and XYP1-18-1, respectively

XYP1, as a novel anti-*T. gondii* polypeptide, showed good anti-*T. gondii* activity [13]. To further reduce the cytotoxicity and synthetic cost of XYP1, bioinformatic analyses and structural modifications were performed to optimize the structure and function of XYP1. On the basis of the predicted tertiary structure of XYP1 (KIKWFKAMKSIAKFIAKDQLKKHL, Fig. 1A), the α-helix structure of the N-terminal was retained and truncated from the C-terminal, resulting in four truncated peptides: XYP1-15, XYP1-16, XYP1-17, and XYP1-18 (Fig. 1). The structure with hydrophobic and hydrophilic residues distributed on both sides is beneficial for enhancing the activity of antimicrobial peptides (AMPs). Therefore, according to the helical wheel projection diagrams of the truncated peptides, the amino acid substitutions were performed on the four peptides. Specifically, leucine (Leu, L) instead of lysine (Lys, K), K instead of isoleucine (Ile, I), K instead of alanine (Ala, A), serine (Ser, S) was replaced with K, phenylalanine (Phe, F) was replaced with lysine (K), and aspartic acid (Asp, D) was replaced with leucine (L). As shown in Fig. 1, four peptides were eventually generated: XYP1-15-1 (LKKWFKKMKKIAKKI), XYP1-16-1 (LKKWFKKMKKIAKKIA), XYP1-17-1 (LKKWFKKMKKIAKKIAK), and XYP1-18-1 (LKKWFKKMKKIAKKIAKL). The properties and parameters of XYP1 and its derived peptides were shown in Additional file 1: Table S1. The high-purity polypeptides were successfully synthesized by solid-phase synthesis. The results of ESI–MS (Additional file 1: Figs. S1 and S2) showed that the actual molecular weight of each polypeptide was consistent with the theoretical molecular weight.

### The derived peptides XYP1-17, XYP1-18, and XYP1-18-1 exhibited anti-*T. gondii* activity

**Fig. 2:**
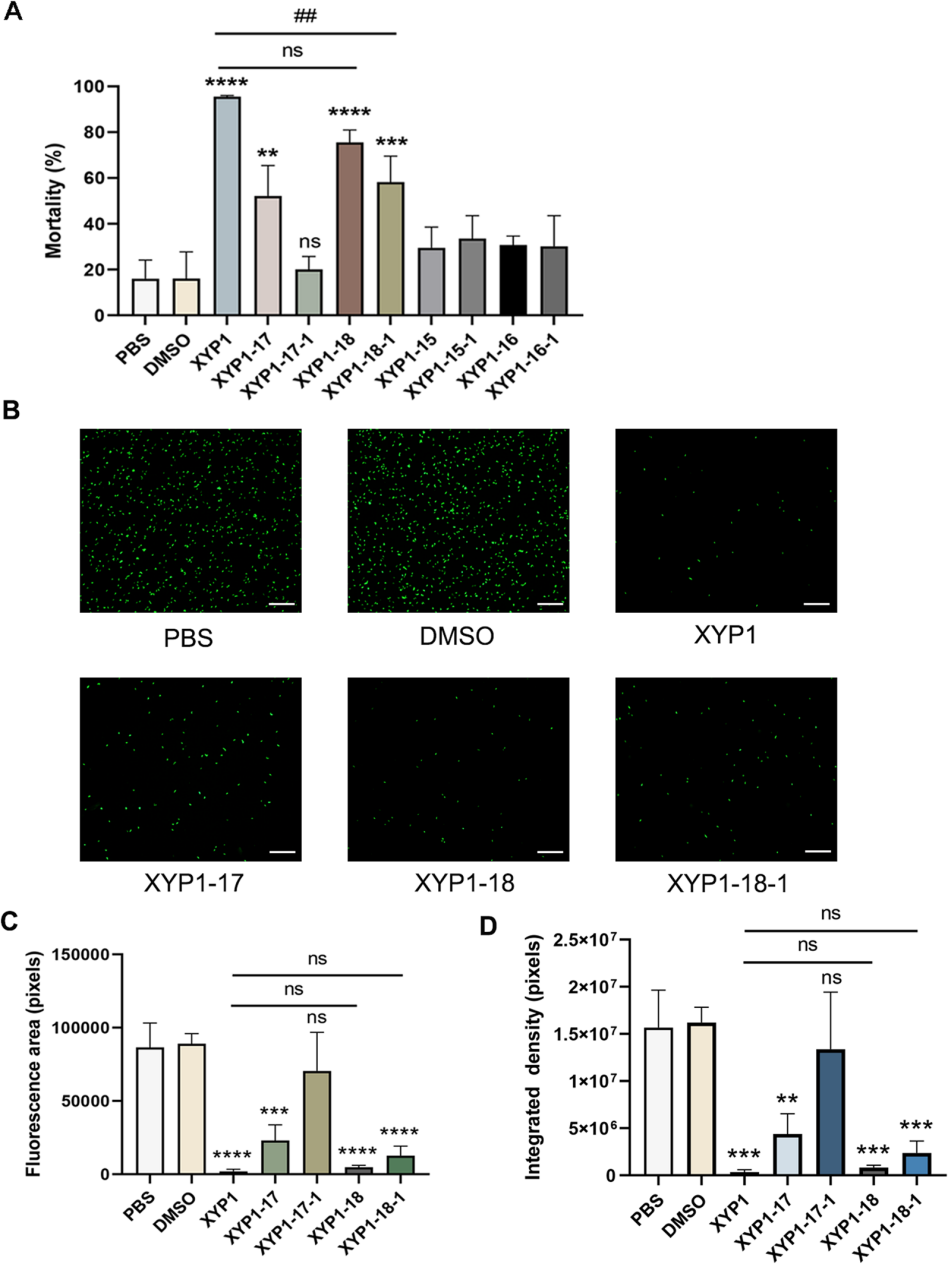
Inhibitory effect of XYP1 and its derived peptides on *T. gondii* in vitro. **A** Statistical diagram of toxoplasma mortality in each group in trypan blue experiment, the killing effect of PBS (negative control group), DMSO (negative control group), and XYP1 and its derived peptides (experimental group) on *T. gondii* at a concentration of 10 μM. *P < 0.05, **P < 0.01, ^##^*P* < 0.01, ***P < 0.001, ****P < 0.0001, “ns” shows *P* > 0.05. **B** Fluorescence microscopy results of control group and XYP1 and derived peptides (XYP1-17, XYP1-18, XYP1-18-1), scale = 100 μm. **C–D** Statistical analysis of fluorescence area and fluorescence intensity of each group, *P < 0.05, **P < 0.01, ***P < 0.001, ****P < 0.0001, “ns” shows *P* > 0.05

Our previous study has confirmed that 10 μM XYP1 can kill *T. gondii* tachyzoites. Therefore, trypan blue staining assay was used to compare the killing activity in vitro of 10 μM different derived peptides and XYP1. The result of XYP1 was consistent with the previous findings. Among these derived peptides, XYP1-17, XYP1-18, and XYP1-18-1 also exhibited good anti-*T. gondii* activity, with XYP1-18 being comparable to XYP1 (Fig. 2A). To further demonstrate the anti-*T. gondii* activity of XYP1-17, XYP1-17-1, XYP1-18, and XYP1-18-1, the tachyzoites of RH-GFP-TgAtg8 *T. gondii* strain were co-incubated with 10 μM concentrations of these peptides. After a 2-h incubation, we observed their anti-*T. gondii* activity through fluorescence microscopy (Fig. 2B). The fluorescence area and intensity of XYP1-17, XYP1-18, and XYP1-18-1 groups were significantly reduced compared with the control group (Fig. 2C, D). These results were consistent with the trypan blue staining assay.

**Fig. 3:**
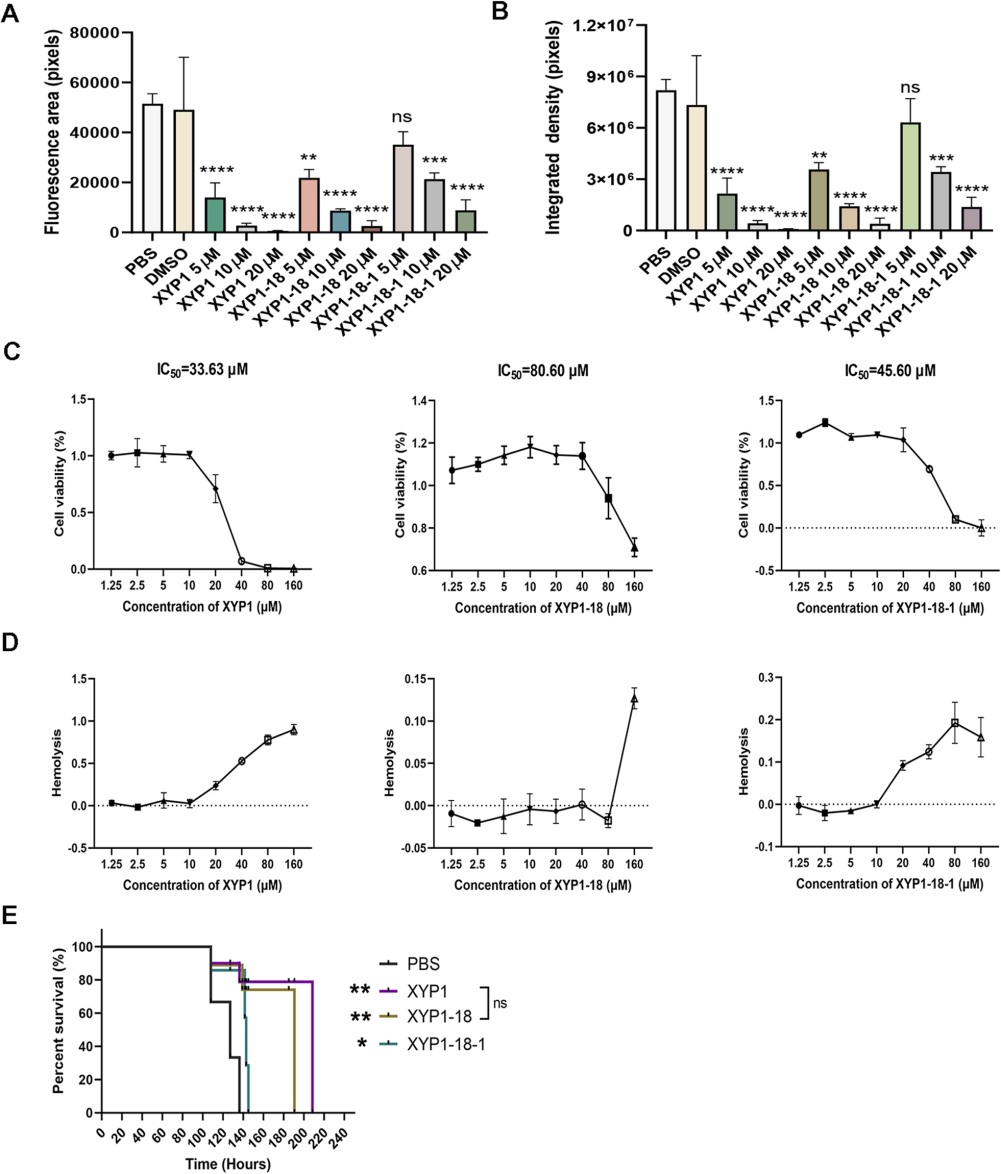
Safety assessment and survival experiments of XYP1-derived peptides. **A**, **B** The killing effect of derived peptides on *T. gondii* at different concentrations; Fig. A is the statistical analysis of fluorescence area of each group, Fig. B is the statistical analysis of fluorescence intensity of each group. *P < 0.05, **P < 0.01, ***P < 0.001, ****P < 0.0001, “ns” shows *P* > 0.05. **C** Cytotoxic effects of XYP1, XYP1-18, and XYP1-18-1 at different concentrations on HFFs; each graph shows the IC_50_ value of each polypeptide to the HFFs. **D** Hemolytic activity of XYP1, XYP1-18, and XYP1-18-1 polypeptides on human red blood cells, hemolysis rate is a measure of hemolytic activity. **E** Survival of mice infected with *T. gondii* after treatment with three peptides: XYP1, XYP1-18, and XYP1-18-1, all drugs were administered to mice at a dose of 4 mg/kg, *P < 0.05, **P < 0.01, ***P < 0.001, ****P < 0.0001, “ns” shows *P* > 0.05

To explore the concentration dependence of the polypeptides against *T. gondii*, XYP1-derived peptide solutions with different concentration gradients (5 μM, 10 μM, 20 μM) were prepared and then mixed with *T. gondii* tachyzoites at room temperature. After incubation for 2 h, the anti-*T. gondii* effect was observed by fluorescence microscopy. The results showed that the killing effect of these three peptides on tachyzoites of RH-GFP-TgAtg8 strain was enhanced with increasing concentration (Fig. 3A), and the fluorescence density of *T. gondii* became weaker (Fig. 3B). Compared with the control group, the fluorescence intensity and fluorescence area of XYP1 and XYP1-18 at different concentrations were statistically different.

### The security of XYP1-18 and XYP1-18-1 was higher than XYP1

According to the results of trypan blue staining and fluorescence microscopy, the peptides XYP1-18 and XYP1-18-1 had better anti-*T. gondii* effects. Therefore, these two peptides were selected for the cytotoxicity test. It was observed that the higher the concentration of XYP1, XYP1-18, and XYP1-18-1, the greater the toxicity to cells (Fig. 3C). The IC_50_ values of XYP1, XYP1-18, and XYP1-18-1 were 33.63 μM, 80.60 μM, and 45.60 μM, respectively. The IC_50_ values of XYP1-18 and XYP1-18-1 were higher than XYP1, indicating higher safety of these two derived peptides.

Hemolysis assay was also used to evaluate the toxic effects of peptides on the host. When XYP1 concentrations ranged from 1.25 μM to 10 μM, almost no hemolysis reaction was observed. However, as the concentration increased, the hemolysis rate gradually increased (Fig. 3D). In contrast, XYP1-18 caused almost no hemolysis at concentrations ranging from 1.25 μM to 80 μM, and only minor hemolysis occurred even at the highest concentration (160 μM). XYP1-18-1 had low hemolysis at 1.25 μM to 160 μM, with a maximum hemolysis rate of only 20% (Fig. 3D). It was indicated that XYP1-18 and XYP1-18-1 were safer than XYP1 over a wider range of concentrations.

### The efficacy of XYP1-18 in vivo was comparable to XYP1

The survival experiment was used to evaluate the therapeutic efficacy of several peptides against *T. gondii* infection in vivo. All mice in the control group died at 127.1 h. In contrast, XYP1 can significantly prolong the survival of mice to 208.5 h, XYP1-18 can be extended to 191 h, and XYP1-18-1 can only be extended to 145 h (Fig. 3E). There is no significant difference between XYP1-18 and XYP1. XYP1-18-1 group had poor effect on prolonging the survival time of mice.

### XYP1-18 and XYP1-18-1 can destroy the surface structure and organelles of *T. gondii*

**Fig. 4:**
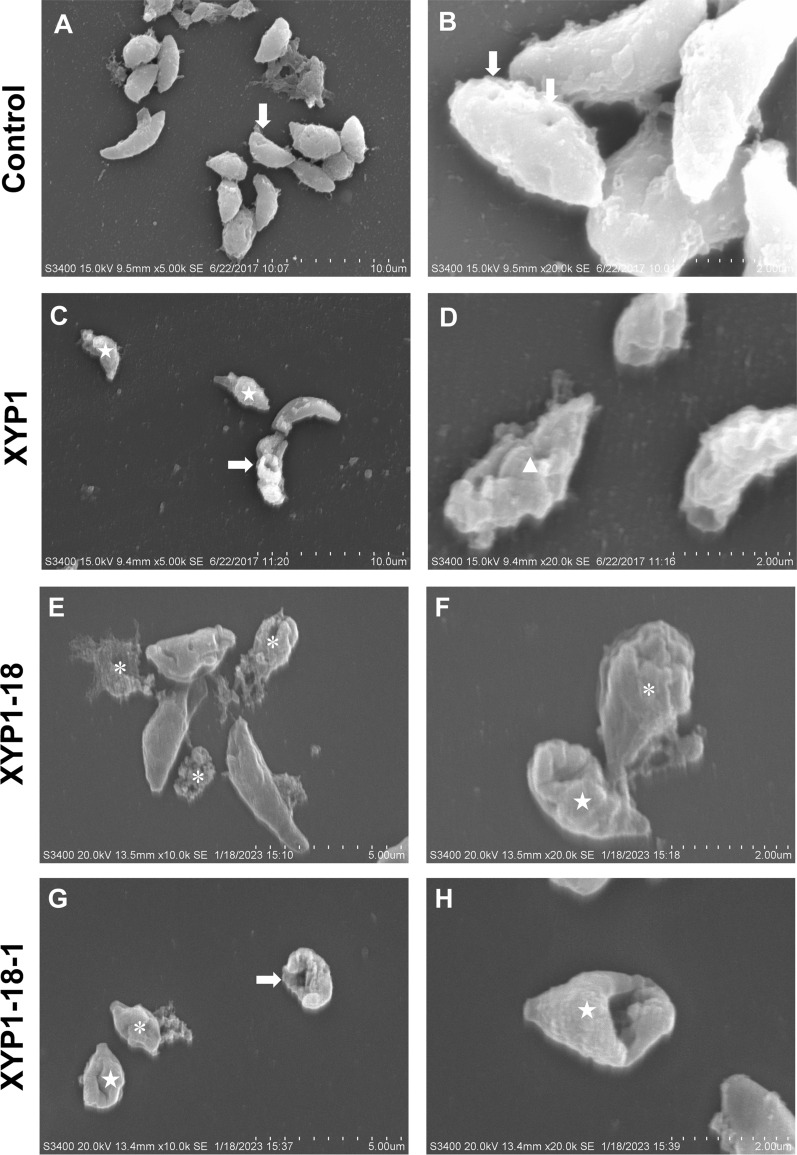
The SEM results of XYP1, XYP1-18, and XYP1-18-1 effects on the surface structure of *T. gondii* tachyzoites. **A**, **B** *T. gondii* tachyzoites were incubated with PBS buffer for 2 h. **C**, **D** *T. gondii* tachyzoites were incubated with XYP1 (10 μM) solution for 2 h. **E**, **F** *T. gondii* tachyzoites were incubated with XYP1-18 (10 μM) solution for 2 h. **G**, **H** *T. gondii* tachyzoites were incubated with XYP1-18-1 (10 μM) solution for 2 h. ↓: micropore; → : holes; ☆: shrunken or sunken tachyzoites of *T. gondii*; *: disintegrating *T. gondii* tachyzoites; enlarged tachyzoite. Scale: **A** = 10 μm; **B** = 2 μm; **C** = 10 μm; **D** = 2 μm; **E** = 5 μm; **F** = 2 μm; **G** = 5 μm; **H** = 2 μm

The effects of XYP1, XYP1-18, and XYP1-18-1 on the surface structure of *T. gondii* tachyzoites were observed using SEM. Normal *T. gondii* tachyzoites were crescent-shaped, with a sharp front end and a rounded back end (Fig. 4A, B). The cell membrane was complete and there were many micropores on the surface. After being treated with 10 μM polypeptides for 2 h, various damages were observed on the surface membranes of tachyzoites. In the XYP1 group, pores formed on the cell membrane surface, and many tachyzoites occurred wrinkling or expansion of the membrane (Fig. 4C, D). Under the influence of XYP1-18, many tachyzoites disintegrated, with depressions appearing on the cell membrane surface (Fig. 4E, F). XYP1-18-1 caused the pores or depressions on the surface of the *T. gondii* cell membranes, and some tachyzoites disintegrated (Fig. 4G, H). These results indicated that the three peptides had varying degrees of impact on the surface morphology of *T. gondii*. Specifically, XYP1-18 and XYP1-18-1 had more pronounced destructive effects on *T. gondii* than XYP1.

**Fig. 5:**
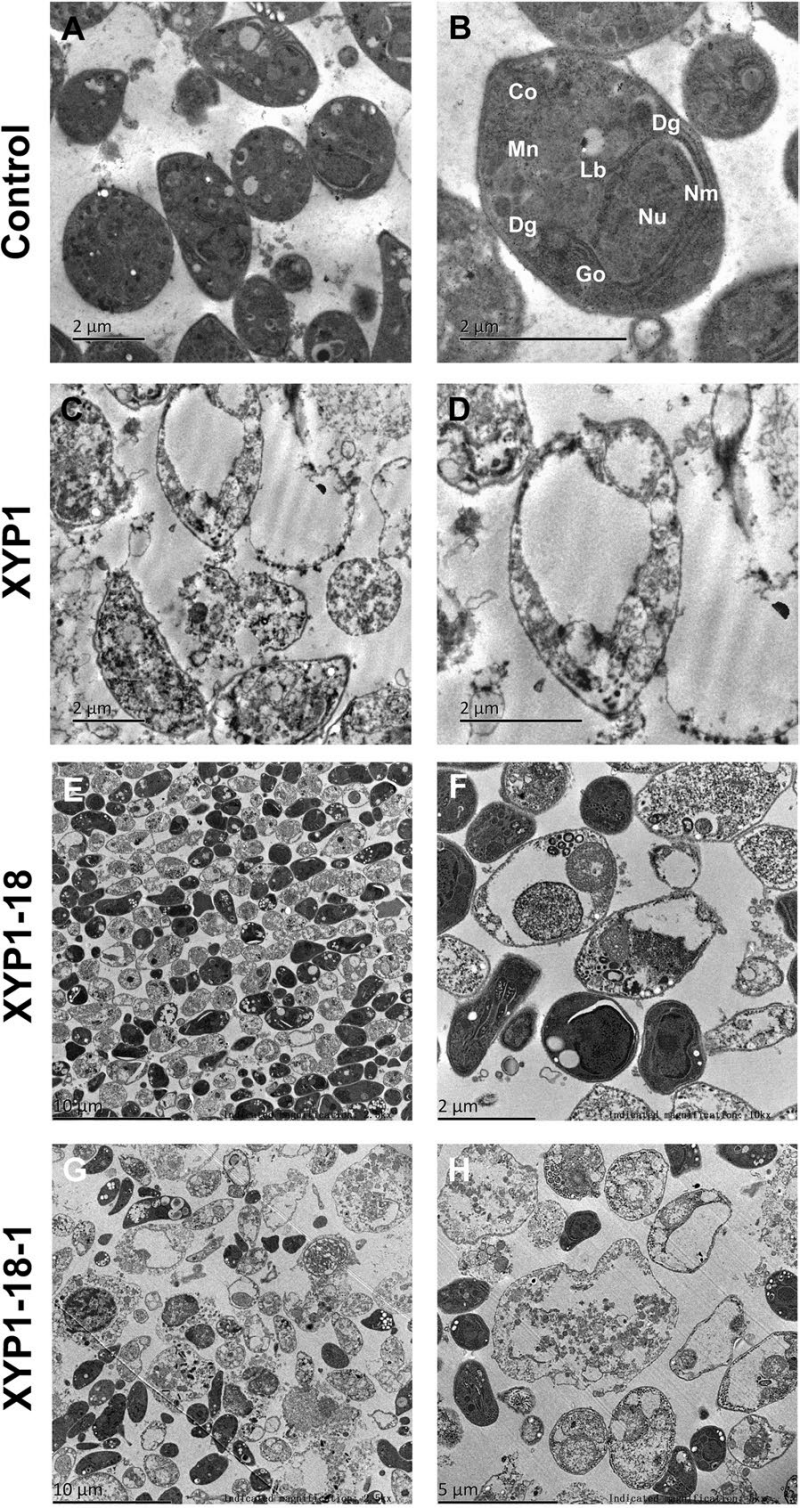
The TEM results of XYP1, XYP1-18, and XYP1-18-1 effects on the internal structure of tachyzoites of *T. gondii.* **A**, **B** *T. gondii* tachyzoites were incubated with PBS buffer for 2 h. **C**, **D** *T. gondii* tachyzoites were incubated with XYP1 (10 μM) solution for 2 h. **E**, **F** *T. gondii* tachyzoites were incubated with XYP1-18 (10 μM) solution for 2 h. **G**, **H** *T. gondii* tachyzoites were incubated with XYP1-18-1 (10 μM) solution for 2 h. Co: conoid; Dg: electron-dense granule; Go: Golgi complex; Lb: lipid body; Nu: nucleus; Nm: nuclear membrane. Scale: **A** = 2 μm; **B** = 2 μm; **C** = 2 μm; **D** = 2 μm; **E** = 10 μm; **F** = 2 μm; **G** = 10 μm; **H** = 5 μm

TEM was used to observe the organelle structure inside *T. gondii*. The organelles inside the untreated tachyzoites were intact (Fig. 5A, B). In the XYP1 group, the tachyzoites not only showed ruptures in the surface membrane, but also different degrees of vacuolization internally (Fig. 5C, D). With the treatment of XYP1-18, the majority of tachyzoites exhibited not only severe deformation and rupture of the surface membrane, but also serious shrinkage and vacuolization internally (Fig. 5E, F). In the XYP1-18-1 group, the internal structure of tachyzoites was even more severely damaged, with not only shrinkage and vacuolization, but also complete dissolution of the tachyzoites observed (Fig. 5G, H). In conclusion, these polypeptides had varying degrees of impact on the internal structure of *T. gondii* tachyzoites.

### XYP1 affected the inflammatory and immune responses of host cells

**Fig. 6:**
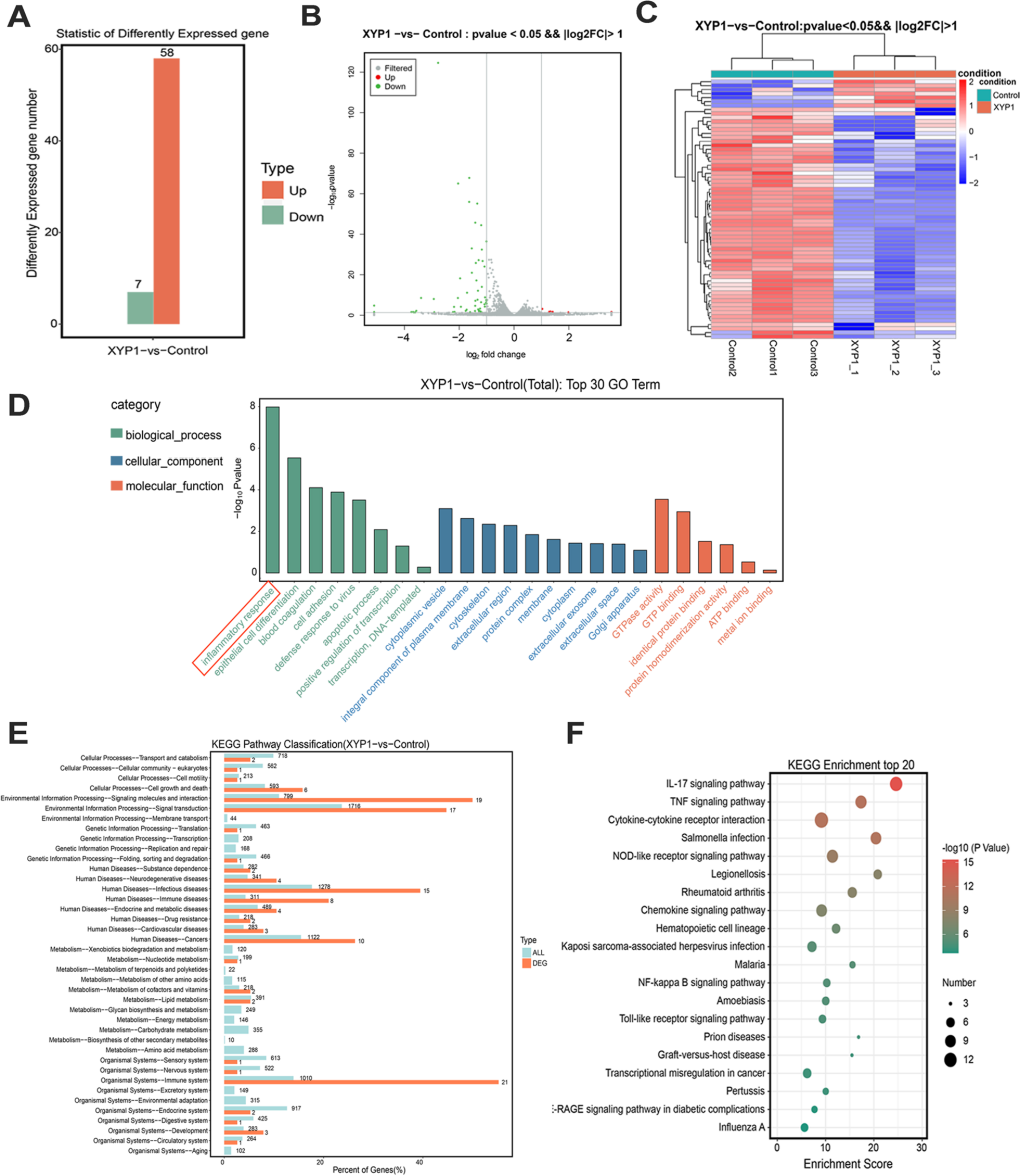
Effects of XYP1 on genes and pathways associated with host cells infected with *T. gondii.* **A** The number of differential genes up-regulated and down-regulated. **B** In the differentially expressed volcano map, green and red dots represent significantly different genes, and gray dots represent nonsignificantly different genes. **C** The results of cluster analysis for each difference group. Red indicates highly expressed genes, blue indicates genes that are low expression. **D** GO enrichment analysis Top30 bar chart. The horizontal coordinate is the GO entry name and the vertical coordinate is −log_10_ *P*-value. **E** Distribution of differentially expressed genes and all genes in KEGG pathway. The horizontal axis is the ratio (%) between the genes annotated to each metabolic pathway and the total number of genes annotated to the KEGG pathway. **F** Bubble diagram of KEGG enrichment Top20, entries with larger bubbles contain more differential protein coding genes, and the redder the bubble color, the greater the difference

Previous studies have initially explored the effects of XYP1 on *T. gondii*-related genes and pathways. To better understand the host cell response to *T. gondii* under the influence of XYP1, changes in genes and pathways before and after XYP1 treatment were analyzed by transcriptome technology. *P* < 0.05 and fold change > 2 were used to determine whether genes in the control group and the XYP1 group showed differential expression. A total of 65 genes had significant differences in expression, including 7 up-regulated genes and 58 down-regulated genes (Fig. 6A). The M-versus-A (MA) map and volcano map represented significantly differential genes under differential screening conditions (Fig. 6B, Additional file 1: Fig. S3A). After clustering analysis of the differentially expressed genes (Fig. 6C), it was observed that the samples from the control group and the XYP1 group clustered together in the same cluster, indicating that these genes exhibited similar expression patterns in the two sample groups and may have similar biological functions.

The 37 differentially expressed genes were classified into 3 main gene ontology (GO) categories, which were further assigned to 64 specific functional subcategories. As shown in Additional file 1: Fig. S3B, a total of 448 significantly enriched GO terms were identified. Among them, 310 terms were enriched in biological process (BP), 65 terms in cellular component (CC), and 73 terms in molecular function (MF). In the top 30 GO terms (Fig. 6D), the most significantly enriched term in BP was “inflammatory response,” in CC it was “vesicle,” and in MF it was “GTPase activity.” The KEGG enrichment results were summarized in Fig. 6E. Immune-related pathways with a higher gene enrichment included the IL-17 signaling pathway, tumor necrosis factor signaling pathway, cytokine-cytokine receptor interaction, NOD(Nucleotide oligomerization domain)-like receptor signaling pathway, and NF-kappa B signaling pathway. The top 20 significantly enriched pathways were selected from the KEGG enrichment results for display in a bubble chart, as shown in Fig. 6F.

**Fig. 7:**
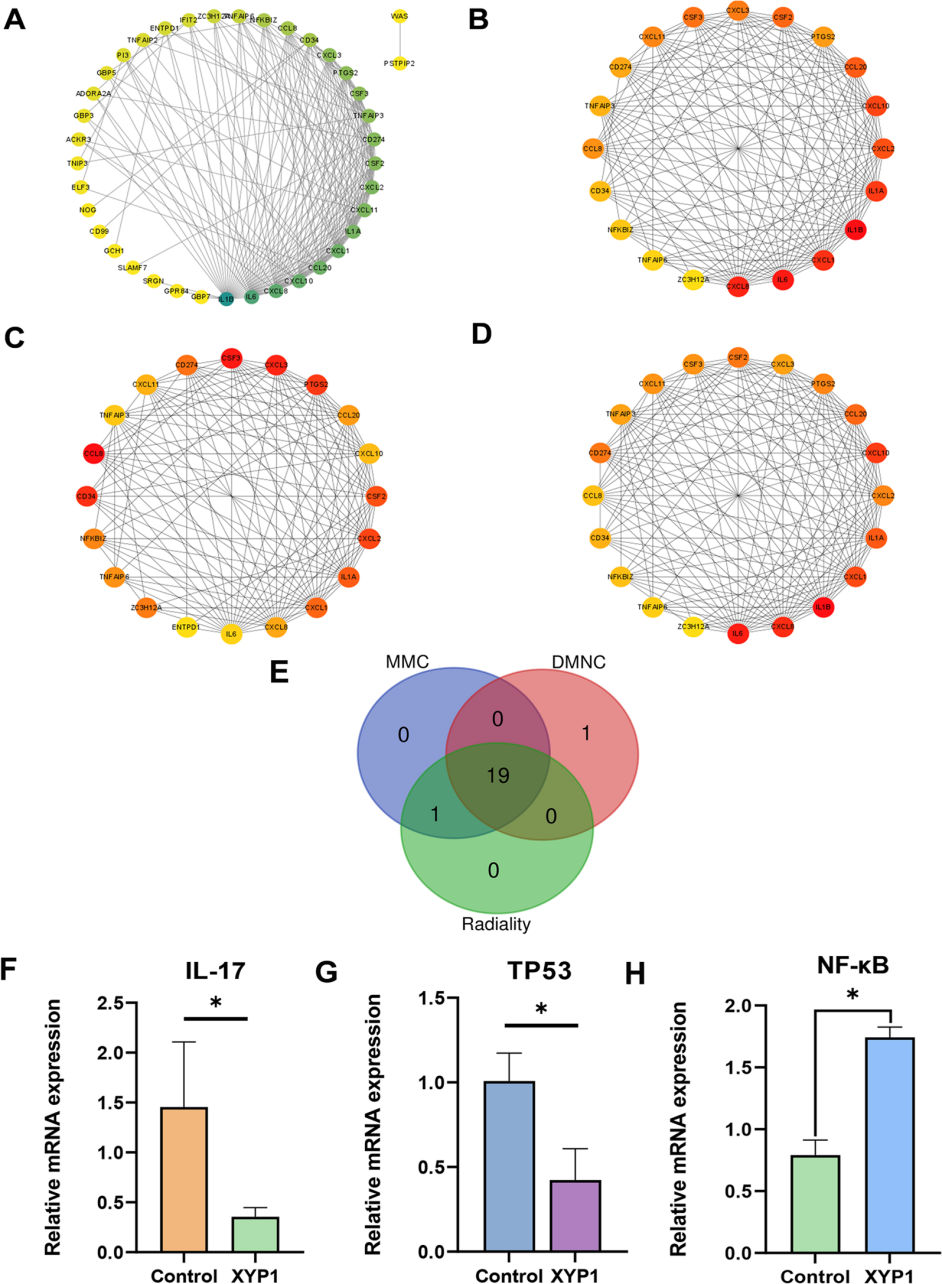
Differential expression protein interaction network analysis and qPCR validation. **A** Circular protein interaction network map of differentially expressed genes, the darker the green color, the higher the node score. **B** The top 20 hub genes based on MMC algorithm. **C** The top 20 hub genes based on DMNC algorithm. **D** The top 20 hub genes based on radiality algorithm. **E** Venn diagram of hub genes intersection of three algorithms. **F**–**H** Changes in transcription levels of *IL-17*, *tp53*, and *NF-κB* genes

Furthermore, protein–protein interaction (PPI) networks were mapped and the core interaction networks were predicted. As shown in Fig. 7A, a darker green color indicates higher score of the nodes. MCC, DMNC, and radiality algorithms are used to screen the Top20 hub proteins (Fig. 7B–D). The redder the color is, the higher the score of the nodes, and the more yellow–green the color is, the lower the score. In the MCC algorithm, the top three proteins were IL1B, IL-6, and CXCL-8; in the DMNC algorithm, the top three proteins were CCL-8, CSF3, and CXCL3; in the radiality algorithm, the top three proteins were IL1B, IL-6, and CXCL-8, consistent with the MCC results. Subsequently, the intersection was taken and a Venn diagram was plotted (Fig. 7E), showing 19 common hub proteins (Additional file 1: Table S2). MMC and radiality shared IL1B. There is ENTPD1 in the DMNC algorithm that has no intersection with the other two algorithms. The results of qPCR showed that the transcription levels of *IL-17* and *TP53* were down-regulated and *NF-kB* was up-regulated in the XYP1 treatment group (Fig. 7F–H).

## Discussion

When *T. gondii* infects humans, it often remains inapparent, but those with congenital infections or compromised immune systems may suffer severe symptoms [34, 35]. The development of novel drugs is a current research focus, and venom peptides derived from animal toxins hold great potential as scaffolds for clinical applications, including cysteine-stabilized peptides and linear helical peptides [36]. In this study, eight derived peptides were successfully generated by amino acid truncation and replacement of XYP1, which originates from the venom of *Lycosa coelestis*. Preliminary results suggested that XYP1 might exert anti-*T. gondii* effect on the basis of an α-helical structure [13]. Phospholipids serve as a major source of negative charge in cell membranes, and active peptides with an α-helical structure often target phospholipids [37]. Therefore, the α-helix structure of the derived peptide is preserved. Because these peptides contain positively charged amino acid side chains, they can interact with phospholipids on the cell membrane by electrostatic interaction. The amphipathic α-helical structure formed by the peptides facilitates their insertion into the cell membrane [38]. When the peptide inserts into the cell membrane, its hydrophobic regions interact with the phospholipid bilayer, disrupting the integrity of the cell membrane, which leads to the formation of pores and gaps. These pores and gaps facilitate the leakage of cellular contents, thereby disrupting cellular homeostasis [39, 40].

Another essential property of AMPs is amphipathy, which refers to the relative abundance of hydrophilic and hydrophobic residues. Studies have shown that indolicidin analogs with increased amphipathy and charge displayed lower hemolytic activity and maintained antimicrobial activity [41, 42]. The distribution of hydrophobic and hydrophilic residues on both sides of the polypeptide plays a crucial role in the biological activity and bioavailability of AMPs [17]. By replacing individual amino acid residues, we successfully generated four new peptides that were structurally similar to truncated peptides but had more evenly distributed hydrophilic and hydrophobic residues, which may enhance their biological activity.

The initial screening experiments revealed that three peptides, XYP1-17, XYP1-18, and XYP1-18-1, exhibited much strong anti-*T. gondii* effects than the control group. Among them, XYP1-18 had similar anti-*T. gondii* activity to XYP1, while the other peptides had minimal effects. The lack of efficiency in XYP1-15 and XYP1-16 could be attributed to the absence of key functional residues induced by a greater number of truncated amino acids. Cationic amino acids, such as lysine (Lys) or arginine (Arg), are crucial for antimicrobial activity in peptide sequences. These common cationic amino acids are typically positioned on the hydrophilic side of the antimicrobial peptides [43–45]. There is a complex interaction among net charge, hydrophobicity, and amphipathicity [46–49]. These parameters play different roles in various peptide sequences, with optimal antimicrobial activity requiring the right balance of hydrophobicity, amphipathicity, and charge density. The truncated XYP1-15 and XYP1-16 significantly increased hydrophobicity and decreased net charge, while excessive hydrophobicity led to increased cytotoxicity and loss of activity, and decreased net charge may lead to decreased activity. Although the amphiphilicity and net charge of XYP1-15-1 and XYP1-16-1 increased, their hydrophobicity decreased, which may affect their anti-*T. gondii* activity.

The main difference between XYP1-17-1 and XYP1-18-1 were the lack of a leucine residue. Typically, higher hydrophobic leucine (Leu, L) can be substituted for lower hydrophobic amino acid residues to compensate for this loss [50–52]. Leucine side chains are relatively large, containing an isopropyl group that contributes to hydrophobic effects. These effects are crucial in protein folding and maintaining the structure of α-helices [53]. Further in vitro screening experiments had shown that XYP1-18 and XYP1-18-1 were the most effective derived-peptides, with lower cytotoxicity and hemolytic activity than XYP1, thus they have better potential as candidates for polypeptide therapy in toxoplasmosis.

We previously discovered that XYP1 can significantly down-regulate the *MIC10*, *HSP29*, and *rpb-10* genes in *T. gondii*, which was associated with membrane component, invasion, and proliferation of *T. gondii*. However, it remains unclear how XYP1 affects the interactions between *T. gondii* and the host. In this study, RNA-seq was used to focus on the differential genes in the host before and after XYP1 treatment. In total, 65 genes showed significant differences in expression, with 7 up-regulated (10.77%) and 58 down-regulated (89.23%) genes. According to GO analysis, the most enriched biological process is the inflammatory response. Genes involved in this process include *ADORA2A*, *NFKBIZ*, *ELF3*, and *TNIP3*. This suggested that XYP1 may eliminate *T. gondii* by modulating the host cell’s immune system to trigger inflammatory response and immune response. When host cells were infected with *T. gondii*, it activated the immune system, leading to the release of inflammatory cytokines such as IL-6, IL-1β, IL-12, and TNF-α. These cytokines promote inflammation, activating the host cell’s immune function to combat *T. gondii* [54–58]. The NF-κB is a driver of inflammation response, and NFKBIZ is an inhibitor of NF-κB [59]. Our findings revealed that XYP1 may kill *T. gondii* by up-regulating the NF-κB regulated pathway. Additionally, on the basis of KEGG analysis, we found that the most significantly enriched pathways among down-regulated genes were the IL-17 signaling pathway, cytokine–cytokine receptor interaction, and TNF signaling pathway. The down-regulation of IL-17 was verified by qRT-PCR assay (Fig. 7F). IL-17 production was associated with protective and pathogenic responses during *T. gondii* infection [60]. Thus, XYP1 might down-regulate the host cell IL-17 signaling pathway, reducing host pathological responses. These pathways were crucial for regulating various biological processes via cell-to-cell interactions and signals.

To sum up, the study of XYP1-derived peptides provided a promising treatment approach for *T. gondii* infection, with great potential for clinical application. While considerable progress has been made, there remain hurdles to overcome. For example, the use of peptide drugs has limitations such as a short half-life. Therefore, future research should focus on improving the properties and effectiveness of peptides to enhance their clinical value. Additionally, further investigation is needed to explore how XYP1 specifically regulates the immune response in host cells to better understand its anti-*T. gondii* mechanisms. In summary, this study lays the groundwork for the development of new anti-*T. gondii* drugs and offers important insights for the research and usage of peptide-based drugs.

## Conclusions

This study successfully identified two derived peptides, XYP1-18 and XYP1-18-1, with anti-*T. gondii* activity through modifying XYP1. In comparison with the parent XYP1, these peptides exhibited lower cost and improved safety (low cytotoxicity and hemolytic activity). Survival assay results demonstrated that XYP1-18-1 also can prolong the survival time of *T. gondii*-infected mice. Furthermore, XYP1 can eliminate *T. gondii* by modulating the inflammatory and immune response of host cells. These discoveries provided a novel strategy and direction for treating *T. gondii* infection, as well as useful references for the development and application of peptide-based therapies.

## Supplementary Information

**Additional file 1: Table S1.** The properties and parameters of XYP1 and its derived peptide. **Table S2.** The hub proteins of intersection of three algorithms. **Figure S1.** The ESI–MS results of XYP1-15, XYP1-15-1, XYP1-16, XYP1-16-1. The abscissa is the mass charge ratio (m/z), and the ordinate is the ionic strength. **Figure S2.** The ESI–MS results of XYP1-17, XYP1-17-1, XYP1-18, XYP1-18-1. **Figure S3.** GO enrichment analysis of differential genes. (A) MA map of differentially expressed genes. The *X*-axis is the normalized average expression of genes in all samples involved in the comparison, and the *Y*-axis is log2Fold Change. Red indicates significant differential genes. (B) Comparison map of differentially expressed genes and distribution of all genes at GO level. Blue represents all the gene-enriched GO entries, red represents the differential gene-enriched GO entries, horizontal axis represents the entry name, and vertical axis represents the number of genes corresponding to the entry and its percentage.

## Data Availability

The HPLC and ESI–MS results of peptides generated during the current study is stored in the Figshare repository, ***:. The RNA-Seq data have been deposited in the Genome Sequence Archive (Genomics, Proteomics & Bioinformatics 2021) in National Genomics Data Center (Nucleic Acids Res 2022), China National Center for Bioinformation/Beijing Institute of Genomics, and Chinese Academy of Sciences (GSA-Human: HRA007277), and are publicly accessible at https://ngdc.cncb.ac.cn/gsa-human.

## Abbreviations

AMP: Antimicrobial peptide
CCL: C-C motif chemokine
CSF: Colony-stimulating factor
CXCL: C-X-C motif chemokine ligand
DMEM: Dulbecco’s modified Eagle’s medium
DMSO: Dimethyl sulfoxide
ESI–MS: Electrospray Ionization mass spectrometry
FBS: Fetal bovine serum
HFF: Human foreskin fibroblast cell
HPLC: High-performance liquid chromatography
IL: Interleukin
OD: Optical density
PBS: Phosphate buffered saline
SEM: Scanning electron microscope
TEM: Transmission electron microscope
TP53: Tumor protein 53
