# Supplementary material for: Anti-Toxoplasma gondii effects of XYP1-derived peptides and regulatory mechanisms of XYP1
Source: Parasit Vectors. 2024 Sep 4;17:376. doi: 10.1186/s13071-024-06455-7 (PMC11373213; doi:10.1186/s13071-024-06455-7)
Supplement: Supplementary file 1 — Additional file 1: Table S1. The properties and parameters of XYP1 and its derived peptide. Table S2. The hub proteins of intersection of three algorithms. Figure S1. The ESI–MS results of XYP1-15, XYP1-15-1, XYP1-16, XYP1-16-1. The abscissa is the mass charge ratio (m/z), and the ordinate is the ionic strength. Figure S2. The ESI–MS results of XYP1-17, XYP1-17-1, XYP1-18, XYP1-18-1. Figure S3. GO enrichment analysis of differential genes. (A) MA map of differentially expressed genes. The X-axis is the normalized average expression of genes in all samples involved in the comparison, and the Y-axis is log2Fold Change. Red indicates significant differential genes. (B) Comparison map of differentially expressed genes and distribution of all genes at GO level. Blue represents all the gene-enriched GO entries, red represents the differential gene-enriched GO entries, horizontal axis represents the entry name, and vertical axis represents the number of genes corresponding to the entry and its percentage. [file 13071_2024_6455_MOESM1_ESM.docx]

Table S1 The properties and parameters of XYP1 and its derived peptide

| Peptide | Sequence | Charge | Isoelectric pH | GRAVY | Hydrophobicity | Instability index |
| --- | --- | --- | --- | --- | --- | --- |
| XYP1 | KIKWFKAMKSIAKFIAKDQLKKHL | +7 | 10.48 | -0.379 | 0.332 | -7.29 |
| XYP1-15 | KIKWFKAMKSIAKFI | +5 | 10.60 | 0.227 | 0.539 | -12.01 |
| XYP1-15-1 | LKKWFKKMKKIAKKI | +8 | 10.85 | -0.853 | 0.197 | 4.07 |
| XYP1-16 | KIKWFKAMKSIAKFIA | +5 | 10.60 | 0.325 | 0.525 | -10.63 |
| XYP1-16-1 | LKKWFKKMKKIAKKIA | +8 | 10.85 | -0.688 | 0.204 | 4.44 |
| XYP1-17 | KIKWFKAMKSIAKFIAK | +6 | 10.70 | 0.076 | 0.436 | -9.24 |
| XYP1-17-1 | LKKWFKKMKKIAKKIAK | +9 | 10.90 | -0.876 | 0.134 | 4.76 |
| XYP1-18 | KIKWFKAMKSIAKFIAKD | +5 | 10.30 | -0.122 | 0.369 | -8.34 |
| XYP1-18-1 | LKKWFKKMKKIAKKIAKL | +9 | 10.90 | -0.617 | 0.221 | 0.34 |

Table S2 The hub proteins of intersection of three algorithms

| Algorithms | Total | Hub protein |
| --- | --- | --- |
| DMNC&MNC&Radiality | 19 | CSF3 /CCL20 /CXCL3/ CXCL8 /CD34 /CXCL2/ CXCL11/ CSF2/  CXCL10 /NFKBIZ /CXCL1 /CCL8 /IL1A/ CD274 /TNFAIP6/ IL6  /PTGS2 /TNFAIP3 /ZC3H12A |
| MMC&Radiality | 1 | IL1B |
| DMNC | 1 | ENTPD1 |


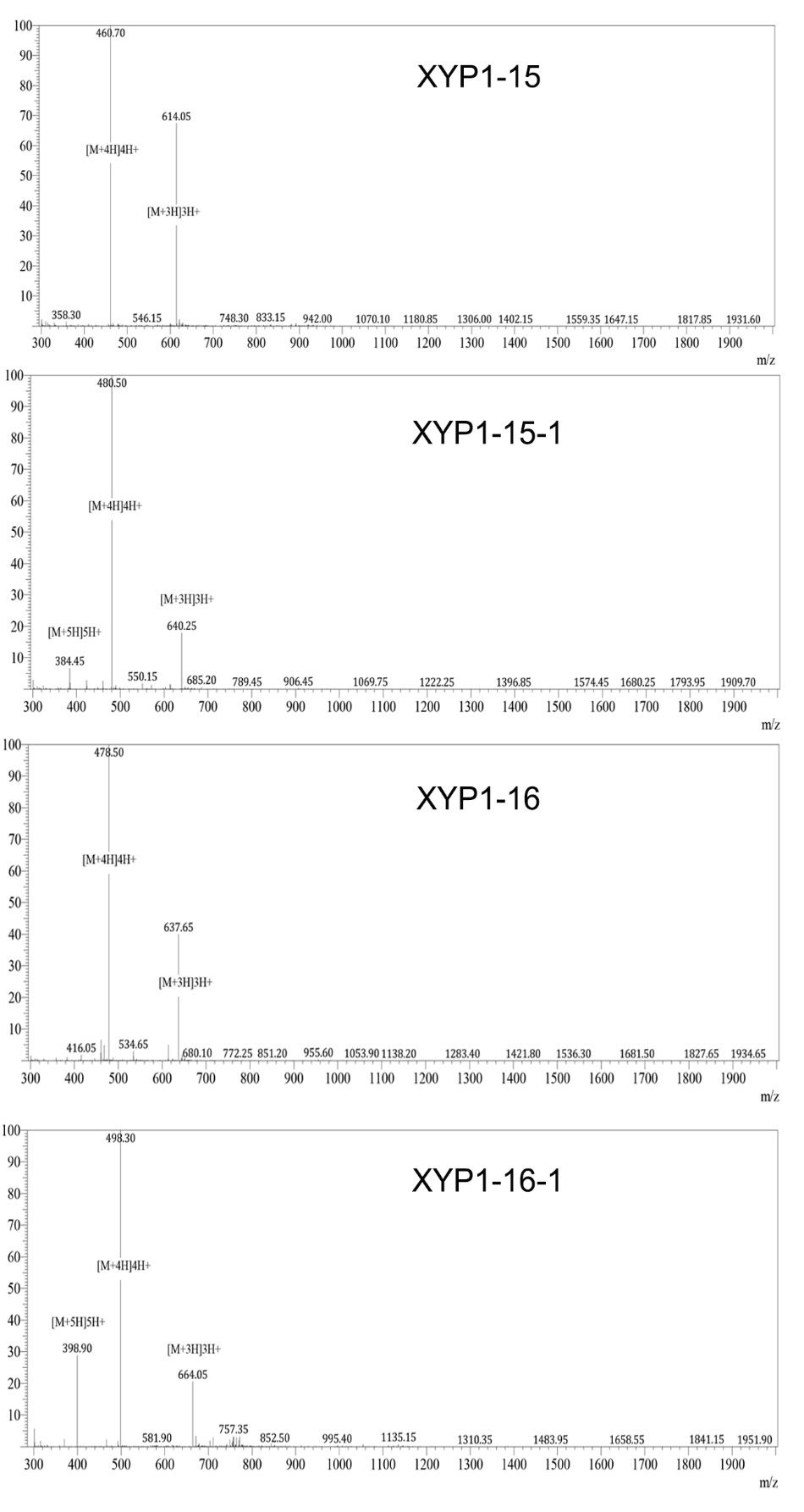


Figure S1 The ESI-MS results of XYP1-15, XYP1-15-1, XYP1-16, XYP1-16-1. The abscissa is the mass charge ratio (m/z), and the ordinate is the ionic strength.


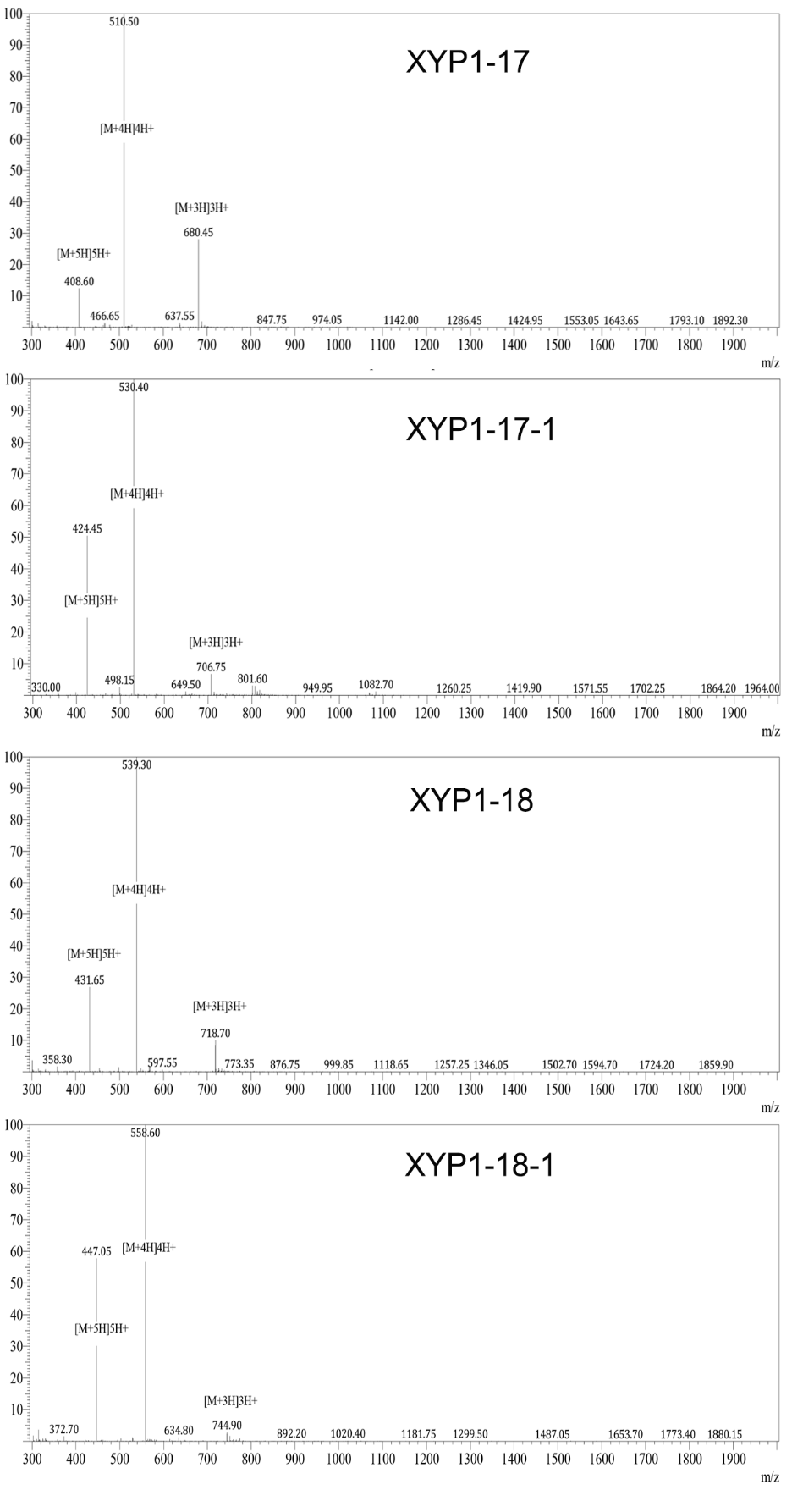


Figure S2 The ESI-MS results of XYP1-17, XYP1-17-1, XYP1-18, XYP1-18-1.


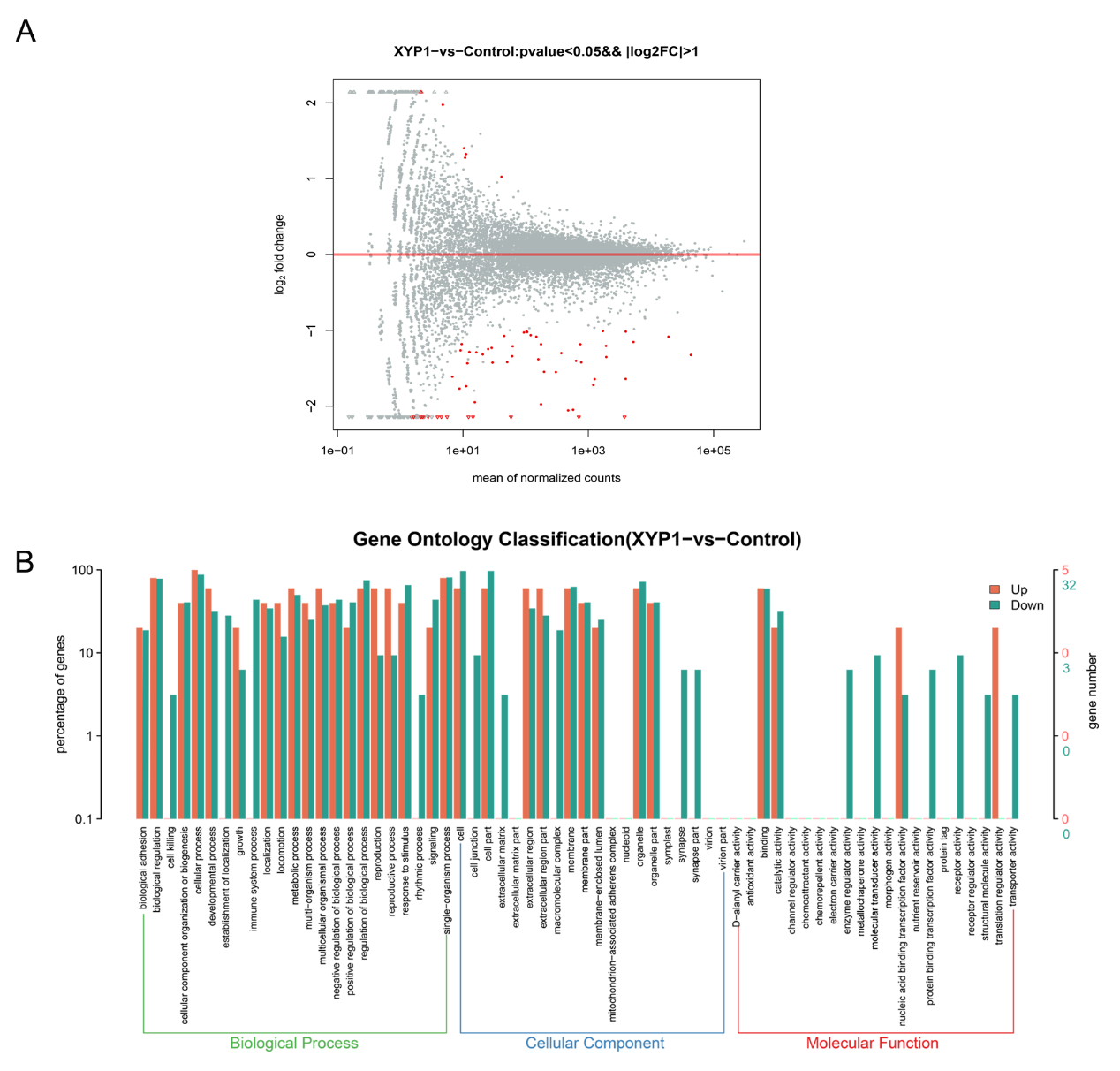


Figure S3 GO enrichment analysis of differential genes. (A) MA map of differentially expressed genes. The X-axis is the normalized average expression of genes in all samples involved in the comparison, and the Y-axis is log_2_Fold Change. Red indicates significant differential genes. (B) Comparison map of differentially expressed genes and distribution of all genes at GO level. Blue represents all the gene enriched GO entries, red represents the differential gene enriched GO entries, horizontal axis represents the entry name, vertical axis represents the number of genes corresponding to the entry and its percentage.
